# Regulatory roles of *Bxy-laf-1* in reproductive behaviour of *Bursaphelenchus xylophilus*

**DOI:** 10.3389/fphys.2022.1024409

**Published:** 2022-11-17

**Authors:** Shimiao Sun, Jinghan Wang, Wenyi Liu, Jing Chen, Lifeng Zhou, Choufei Wu, Hongshi Yu, Jiafu Hu

**Affiliations:** ^1^ College of Forestry and Biotechnology, Zhejiang Agricultural and Forestry University, Hangzhou, China; ^2^ Key Laboratory of Vector Biology and Pathogen Control of Zhejiang Province, School of Life Sciences, Huzhou University, Huzhou, China; ^3^ School of BioSciences, The University of Melbourne, Parkville, VIC, Australia

**Keywords:** *Bursaphelenchus* xylophilus, LAF-1, RNA interference, RT-qPCR, spatial-temporal expression

## Abstract

*Bursaphelenchus xylophilu* is a worldwide quarantine nematode, causing huge economic losses and ecological disasters in many countries. The sex ratio of *B. xylophilus* plays an important role in the nematode infestation. The *laf-1-*related genes are highly conserved in animals, playing crucial roles in sex determination. Therefore, we investigated the expression pattern and biological function of its orthologue, *Bxy-laf-1* in *B. xylophilus*. *Bxy-laf-1* has two typical conserved DNA-binding domains, DEAD and Helicase C. The real-time quantitative PCR data revealed that *Bxy-laf-1* expression was required throughout the entire life of *B. xylophilus*, with the maximum expression in the J2 stage and the lowest expression in the adult stage. mRNA *in situ* hybridization showed that *Bxy-laf-1* is mainly located in the cephalopharynx and reproductive organs of *B. xylophilus*. RNA interference (RNAi) indicated that the head swing frequency was dramatically decreased. The RNA interference results displayed that a significant reduction in motility was observed in the hatched larvae. The female to male sex ratio was also decreased in the F0 and F1 generations, but recovered in the F2 generation. The tail of female adults with eggs in the belly appeared deformities. This phenomenon appeared in the F0 and F1 generations, but recovered in the F2 generation. *Bxy-laf-1* is a typical sex-determination gene with distinct expression patterns in males and females. As demonstrated in other species, the sex ratio was altered after knocking down *Bxy-laf-1* expression. The results of this study contribute to our understanding of the molecular processes of *Bxy-laf-1* in *B. xylophilus*, which may provide clues about how to control pine wilt disease by inhibiting ontogenic growth and reducing nematode fertility.

## 1 Introduction

*Bursaphelenchus xylophilus* (pinewood nematode) is a global quarantine nematode that is originally from the United States (Wingfield et al., 1982) before spreading to Asia and Europe. It has now wreaked havoc on the invading region’s economy (Sun, 1982; Yi et al., 1989; Mota et al., 1999; Kikuchi et al., 2011; Robertson et al., 2011; Zhao et al., 2016). In *B. xylophilus* endemic locations, previous studies have demonstrated significant interspecific conflict between invading nematodes and native sister nematodes (*Bursaphelenchus mucronatus*), with *B. xylophilus* finally displacing native nematodes (Cheng et al., 2009). In recent studies, we discovered that the sex ratio (female-to-male) of the two nematodes increases dramatically in mixed cultures, suggesting that the shift in the sex ratio played a key role in species competition. As a result, altering the sex ratio is a new concept for controlling the pest’s invasion. The fundamental elements that determine the sex of pine worms are still unknown.

The *laf-1* gene is a critical male sex determination gene in *Caenorhabditis elegans* (Haag, 2005), which favors male cell fate by negatively regulating the expression of the *tra-2* gene in hermaphrodites and males, resulting in more male progeny (Hubert and Anderson, 2009). At all stages of development, it is expressed in the somatic cells of male gonads and hermaphrodite nematodes. The *laf-1* heterozygous mutant in *C. elegans* causes XX animals to mature into females and partially feminizes XO somatic cells and germline (Doniach, 1986; Schedl and Kimble, 1988; Goodwin et al., 1997). In addition, *laf-1* is required for embryonic development. A pure mutation in *laf-1* causes the death of embryos or larval in *C. elegans* (Hubert and Anderson, 2009). Its significance in embryo and gonadal development has been confirmed. Therefore, it assumes that *laf-1* may play a conserved role in determining sex fate in *B. xylophilus.* Regulating sex ratios in nematode populations may provide a promising clue for managing nematodes. However, no research on *Bxy-laf-1* has been reported in *B. xylophilus* yet.

*Bxy-laf-1* gene was cloned to investigate the expression pattern and biological function in *B. xylophilus*. In this work, sequence alignment, phylogenetic analysis and 3D-struction predication were performed and real-time quantitative PCR (RT-qPCR), whole-mount *in situ* hybridization and RNA interference (RNAi) were used to examine the expression levels at different stages of *B. xylophilus* development and biological functions of *Bxy-laf-1*. This is the first study to show that *Bxy-laf-1* is required for the mobility, early development, reproduction and mating behavior of *B. xylophilus*.

## 2 Materials and methods

### 2.1 Nematode collection

The *B. xylophilus* NXY61 nematode population was received from the Chinese Academy of Forestry’s Research Institute of Forest Protection in Beijing, China, and was first isolated from sick Pinus massoniana in Ningbo, Zhejiang province, China. *Botrytis cinerea*, a fungal food source, was supplied by a Zhejiang University laboratory. Nematodes were cultivated in Petri dishes with potato dextrose agar (PDA) media at 25°C in the dark on a lawn of *B. cinerea* (Mamiya, 1975; Wang, 2007).

The isolated mixed stage nematodes were transferred to a fresh Petri dish containing sterile distilled water and allowed to lay embryos for 1 h at 25°C in the dark to collect synchronized *B. xylophilus* samples (Futai, 1980). The synchronized embryos were collected after the mixed stage nematodes were removed. The recovered synchronous embryos were cultured for 24 h in the dark at 25°C to produce synchronous second-stage juveniles (J2) (Wang et al., 2005). Following that, the J2 larvae were centrifuged at 3,000 rpm for 3 min, the supernatant was removed, and they were put on *B. cinerea* for 29, 48, and 72 h at 25°C in the dark, separately (Zhu et al., 2016). The Bellman funnel method was used to capture synchronized juveniles from J2 to the fourth stage (J4) and adults (Viglierchio and Schmitt, 1983).

### 2.2 *Bxy-laf-1* gene cloning

TRIZOL Reagent (Thermo Fisher Scientific, China) was used to extract total RNA from *B. xylophilus* at various developmental stages, according to the manufacturer’s instructions. After that, the RNAs were processed with RNase-free DNase I. (Thermo Fisher Scientific). A NanoDrop^®^ ND-2000 spectrophotometer was used to determine RNA concentration and purity (Thermo Fisher Scientific). By reverse transcription with PrimeScript™ RT reagent Kit with gDNA Eraser, first-strand cDNA was generated from 0.5 g of total RNA using a combination of oligo dT primers and random hexamers (Takara, China).

To amplify *laf-1* in *B. xylophilus*, a set of specialized primers (Table 1) were created. 100 ng of cDNA, 10 μL 2 × Ex Taq Polymerase Premix (TaKaRa Biotechnology Co. Ltd., Dalian, China), 1 μL of each primer (10 pmol L^−1^), and sterile distilled water were used in a 20 μL reaction. The following was the amplification procedure: 5 min at 94°C, then 35 cycles of 30 s at 94°C, 30 s at 60°C, and 1 min at 72°C, followed by a 5-min extension step at 72°C. The amplified products were then cloned into a pGEM®-T Easy vector for sequencing (Promega, Madison, WI, United States).

**TABLE 1:** Primers used in the present study.

Primer	Gene symbol	Sequence (5′ to 3′)
Cloning	*Bxy-laf-1*	F: ACC​CCA​ATT​ACG​TCG​GCT​TT
		R: GGC​TGA​TTC​TGG​AAG​CCA​CT
RT-qPCR	*Bxy-laf-1*	F: GGTCGTTTGATTGATGTG
		R: CCTTCGGGAATGTAGCC
	*tbb-2*	F: GAA​TCC​AAC​ATG​AAC​GAT​CT
		R: CTT​CGT​ATT​CAC​CAT​CAT​CT
	*β-actin*	F: CGC​AAA​TAC​TCC​GTC​TGG​ATT​GG
		R: TTC​GTC​GTA​CTC​TTG​CTT​GGA​GA
Synthesis of dsRNA	*Bxy-laf-1*	F:TAATACGACTCACTATAGGGACCCCAATTACGTCGGCTTT
		R:TAATACGACTCACTATAGGGGGCTGATTCTGGAAGCCACT
	*gfp*	F:TAATACGACTCACTATAGGGAAAGGAGAAGAACTTTTCAC
		R:TAATACGACTCACTATAGGGCTGTTACAAACTCAAGAAGG

Italic values represent primers used in cloning, RT-qPCR and synthesis of dsRNA.

### 2.3 Sequence analysis of *Bxy-laf-1*

To analyze the sequence of *Bxy-laf-1*, the cloned fragment obtained *via* the above process was used to BLAST our recently sequenced transcriptome. Additional protein sequences for alignments were obtained from Genbank (www.ncbi.nlm.nih.gov/genbank/). DNAMAN software (Lynnon Biosoft) was used to perform multiple sequence alignments, and MEGAX was used to create a phylogenetic tree using the neighbour-joining technique (Sudhir et al., 2018). The nucleotide sequences of cloned genes were used to determine the protein sequences of *Bxy-laf-1* of *B. xylophilus*. Genbank (www.ncbi.nlm.nih.gov/genbank/) was also utilized to get protein sequences for additional species used in alignments. TMHMM server 2.0 (http://www.cbs.dtu.dk/services/TMHMM/) and SignaIP 5.0 (http://www.cbs.dtu.dk/services/SignaIP/) were used to predict *Bxy-laf-1* transmembrane helices and signal sequences, respectively. ExPASy ProtParam (https://web.expasy.org/protparam/) was used to evaluate the physicochemical parameters of the inferred amino acid sequence of *Bxy-laf-1*, such as amino acid composition, molecular weight, isoelectric point, and instability (Gasteiger et al., 2003).

### 2.4 *Bxy-laf-1* protein modeling

The predicted three-dimensional structure of Bxy-LAF-1 was modeled with AlphaFold2 (John et al., 2021). The structure visualization was created in PyMOL program (Schrödinger, LLC) (Rigsby and Parker, 2016).

### 2.5 *Bxy-laf-1* RT-qPCR analysis

RT-qPCR technique was used to examine the expression profile of *Bxy-laf-1* in *B. xylophilus* at various developmental stages (embryo, J2, J3, J4, and adult). The same procedure was used to extract total RNA and convert it to cDNA as previously reported. The qTOWER 2.2 qPCR System (Analytik JenaAG) was used to perform RT-qPCR with TB Green^®^ Premix Ex Taq IITM (TaKaRa, TliRNaseH Plus). Internal loading controls included the stable reference genes *β-actin* (GenBank accession number EU100952.1) and *tbb-2* (*tubulin beta-2 chain*, GenBank accession number MT769316). The 2^−ΔΔCT^ approach was used to determine the relative expression level of *Bxy-laf-1* (Livak and Schmittgen, 2001). Beacon Designer software was used to create primers for the *Bxy-laf-1* and reference genes, following the manufacturer’s instructions (Table 1) (Brenda and Chhandak, 2015). Melt curves and standard curves were used to confirm the primers’ specificity and amplification efficiency, respectively.

### 2.6 Whole-mount *in situ* hybridization of *Bxy-laf-1*

mRNA *in situ* hybridization was done at different developmental stages (embryo, J2, J3, J4, female adult, and male adult) of *B. xylophilus* to investigate the temporal and spatial expression patterns of *Bxy-laf-1*, based on the procedure of Motohashi et al. in *C. elegans* with minimal modifications (Motohashi et al., 2006; Tang et al., 2020). Probes were made from the cloned fragment (1,085 bp) acquired using the aforementioned procedure. A DIG RNA labeling kit was used to produce and label antisense and sense RNA probes (Roche Gmbh, Mannheim, Germany). A ZEISS Inverted Observer light microscope was used to make the final observations (Carl Zeiss, Germany). As a negative control, the sensing probe was employed.

### 2.7 Synthesis of dsRNA

The roles of *Bxy-laf-1* in *B. xylophilus* were investigated using RNAi. The dsRNA fragments for *Bxy-laf-1* and *gfp* were synthesized using a MEGAscript^®^ T7 High Yield Transcription Kit (Thermo Fisher Scientific Inc.) and the primers as directed by the manufacturer (Table 1). A NanoDrop®ND-2000 spectrophotometer (Thermo Fisher Scientific, United States) was used to assess the quality of produced dsRNAs, which were then visualized in a 1.5% agarose gel.

### 2.8 Assay on locomotion, ontogenesis and sex ratio after RNAi

The RNAi efficiency was determined using specific *Bxy-laf-1* primer sets (Table 1). Three parallel sets of experiments were run, with four replicate wells set up each time, for a total of three biological replicates.

Synchronized embryos and J2 (approximately 20,000 individuals of each stage) were washed and soaked for 24 h in the dark at 25°C in dsRNA (0.5 μg m L^−1^) with the soaking buffer (0.05 percent gelatin, 5.5 mM KH2PO4, 2.1 mM NaCl, 4.7 mM NH4Cl, 3 mM spermidine). As a control, the same number of nematodes were soaked in the soaking buffer without dsRNA or *gfp* dsRNA. Each treatment was replicated three times. Following treatment, the different developmental stages were cultured separately on a *B. cinerea* lawn in the PDA plate to adulthood (F0) at 25°C (Tabara et al., 1998). After they gave birth to F1 embryos, collect 1,000 adult F0 nematodes and the results of sex ratio was statistically analyzed. Then the F1 generation embryos were cultured to adulthood, and the same method was used to collect, count and analyze the sex ratio of the F1 generation and F2 generation adults.

The movement of these nematodes was captured using an Axio Cam Mrm camera and Zeiss Axio Vision software (Carl Zeiss) at 25°C. Thirty-second films were acquired for each position. A successful head thrash was defined as a reorientation of more than 120° in a single head swing. Each replicate used thirty synchronized J2, J3 nematodes, and the experiment was repeated three times. The Nikon NI-SS microscope was used to examine morphological phenotypes (Nikon, Japan). Nikon DS-Ri2 camera was used to capture differential interference contrast (DIC) images.

To explore the influence of *Bxy-laf-1* on progeny production, virgin adults were grown and employed in the study. J2 were soaked in the buffer with dsRNA (0.5 mg L^−1^) and without dsRNA for 24 h to improve mating efficiency and increase the possibilities of mating observation, and J4 was obtained by synchronous culture for 24 h. Synchronized male and female J4 were cultivated separately for 24 h to yield virgin mature males and females. There are four cross mating combinations (CK ♀ + CK ♂, RNAi ♀ + CK ♂, CK ♀ + RNAi ♂, RNAi ♀ + RNAi ♂), CK means control. (Li et al., 2020). Under a microscope, a virgin male and virgin female were placed in a drop of water on a concave glass slide. In a humidifier, the slides were stored in the dark at 25°C (Zhou et al., 2020). A zoom stereomicroscope (Zeiss, Thornwood, United States) was used to investigate the mating behavior of male and female adults. The percentage of error positioning ratio (males who did not correctly locate the female vulva) and average number of offspring was calculated. Each experimental treatment had three sets of 30 pairs of nematodes (30 males and 30 females), and the experiment was performed three times.

### 2.9 Statistics

All data, including head thrashing frequency, improper positioning ratio, and sex ratios, were presented as mean standard error. SPSS 22.0 was used to do statistical analysis and calculations based on the independent sample *t*-test and one-way ANOVA (SPSS Inc., United States). The results were deemed statistically significant when *p* < 0.05 was used. All RT-qPCRs were performed in triplicate.

## 3 Results

### 3.1 Sequence analysis of *Bxy-laf-1*

**FIGURE 1:**
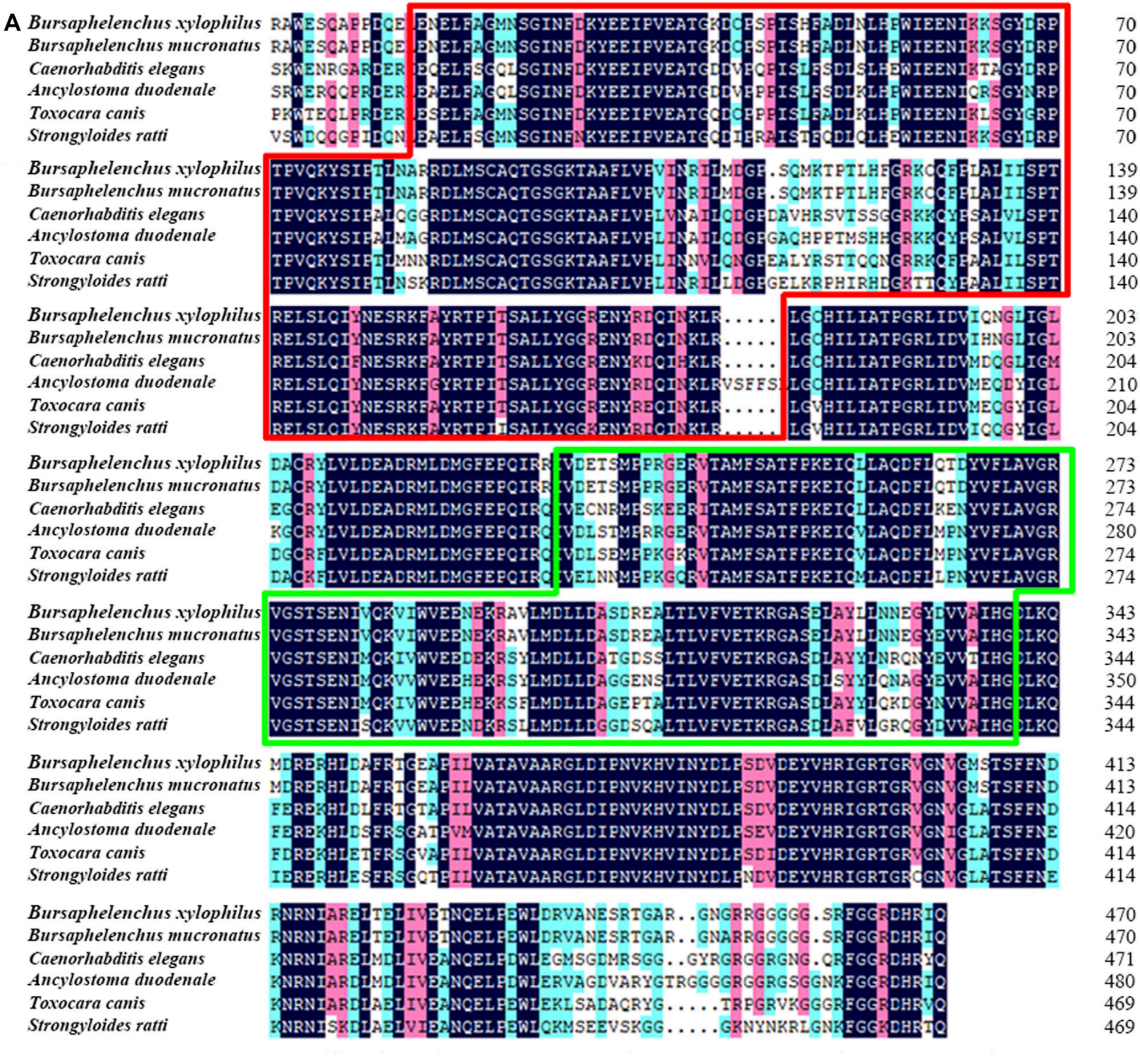
Sequence analysis of *Bxy-laf-1.* **(A)** Multiple amino acid sequence alignments. *B. xylophilus* (CAD5234552.1), *B. mucronatus* (OM210018), *Caenorhabditis elegans* (NP 001254859.1), *Ancylostoma duodenale* (KIH65785.1), *Toxocara canis* (KHN84719.1) and *Strongyloides ratti* (XP 024501163.1). Black, red and blue shading indicates invariant, highly conserved and moderately conserved amino acid residues, respectively. The two conserved DNA-binding motifs of LAF-1, DEAD and Helicase C, are enclosed by the red and green boxes, respectively.

**FIGURE 2:**
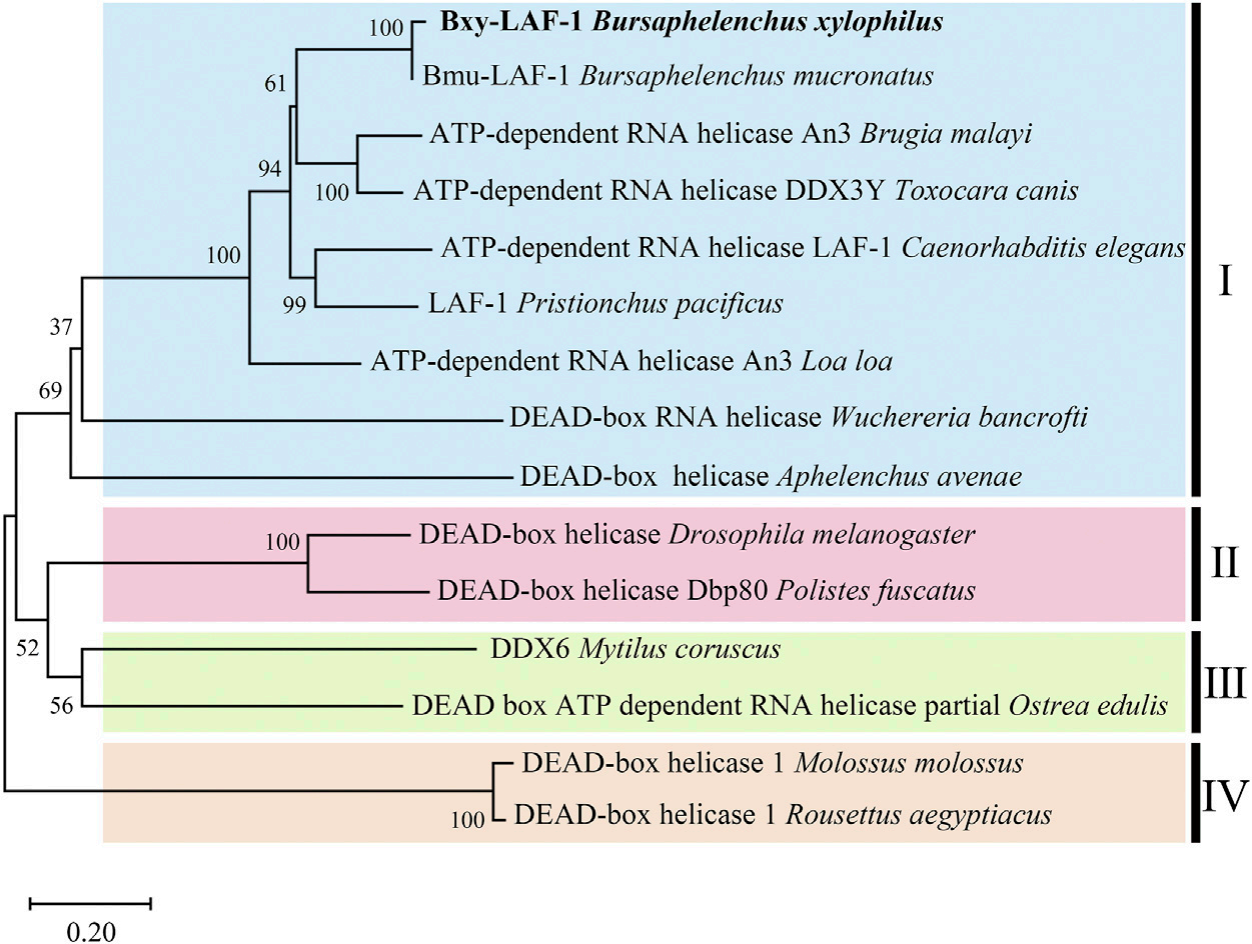
Phylogenetic analysis of LAF-1s from different species. The scale bar indicates evolutionary distance (0.20 substitutions per position). Ⅰ (LAF-1s in Nematoda): *Bursaphelenchus xylophilus* (CAD5234552.1), *Bursaphelenchus mucronatus* (OM210018), *Brugia malayi* (XP 042930654.1), *Toxocara canis* (KHN84719.1), *Caenorhabditis elegans* (NP 001254859.1), *Pristionchus pacificus* (KAF8381410.1), *Loa loa* (XP 003140162.1), *Wuchereria bancrofti* (EJW85565.1), *Aphelenchus avenae* (KAH7731651.1); Ⅱ (LAF-1s in Arthropoda): *Drosophila melanogaster* (AAC23709.1), *Polistes fuscatus* (XP 043504272.1); Ⅲ (LAF-1s in Molluscs): *Mytilus coruscus* (CAC5359041.1), *Ostrea edulis* (AFJ91775.1); Ⅳ (LAF-1s in Chordata): *Molossus* (KAF6429176.1), *Rousettus aegyptiacus* (KAF6446427.1).

Full length of the orthologue of *laf-1, Bxy-laf-1,* was determined, which contained an open reading frame (ORF) of 2,163 bp and encoded 720 amino acids in *B xylophilus*. This gene has one copy number and no other isoforms in the genome. The estimated molecular formula of the *Bxy-laf-1* protein was C_3512_H_5548_N_1038_O_1068_S_29_ with a theoretical equivalent pI value of 8.67, a molecular weight of 80.33 kDa and an atomic number of 11,195. It is speculated that *Bxy-laf-1* may be an unstable protein with a calculated instability index (Ⅱ) of 43.75. Protein sequence of LAF-1 alignment clearly revealed that BXY-LAF-1 had two conserved domains, DEAD and Helicase C (Figure 1). These two domains belong to an ATP-dependent RNA helicase required during sperm development and is essential for the integrity of the reproductive system (Lu et al., 2020). In addition, Bxy-LAF-1 of *B. xylophilus* had the highest level of similarities with Bmu-LAF-1 of *B. mucronatus*. To uncover the evolutionary relationship between the *Bxy-laf-1* gene and the *laf-1* gene of other species, the phylogenetic tree of LAF-1 was constructed, showing that Bxy-LAF-1 and Bmu-LAF-1 formed one branch, and further produced a big node with other LAF-1 proteins in nematodes, such as LAF-1 of *C. elegans* (Figure 2).

### 3.2 Molecular modeling of *Bxy-laf-1* protein

**FIGURE 3:**
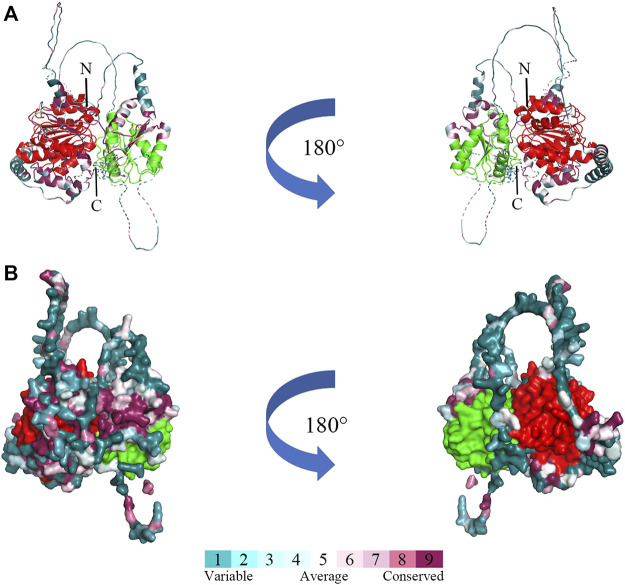
Predicted three-dimensional structure of *Bxy-laf-1*. **(A)** Cartoon representative of Bxy-LAF-1. N, N-terminal; C, C-terminal. **(B)** The Bxy-LAF-1 protein in surface representation. The right model is rotated along the long axis from the left model. The coloring gradient ranges from variable region to conserved regions, and the two conserved DNA-binding motifs are colored by red and green. The red area is DEAD box helicase, and the green is Helicase conserved C-terminal domain.

The three-dimensional structure prediction of *Bxy-laf-1* protein of *B. xylophilus* was modeled by AlphaFold2, using 6KUX as the template (Figure 3). *Bxy-laf-1* exhibits two conserved structural domains, DEAD box helicase and Helicase conserved C-terminal domain (Figure 3A). The molecular surface of *Bxy-laf-1* was also calculated using the PyMOL program (Figure 3B), the variable region and conserved region were colored from blue to purple.

### 3.3 Quantitative analysis of *Bxy-laf-1*

**FIGURE 4:**
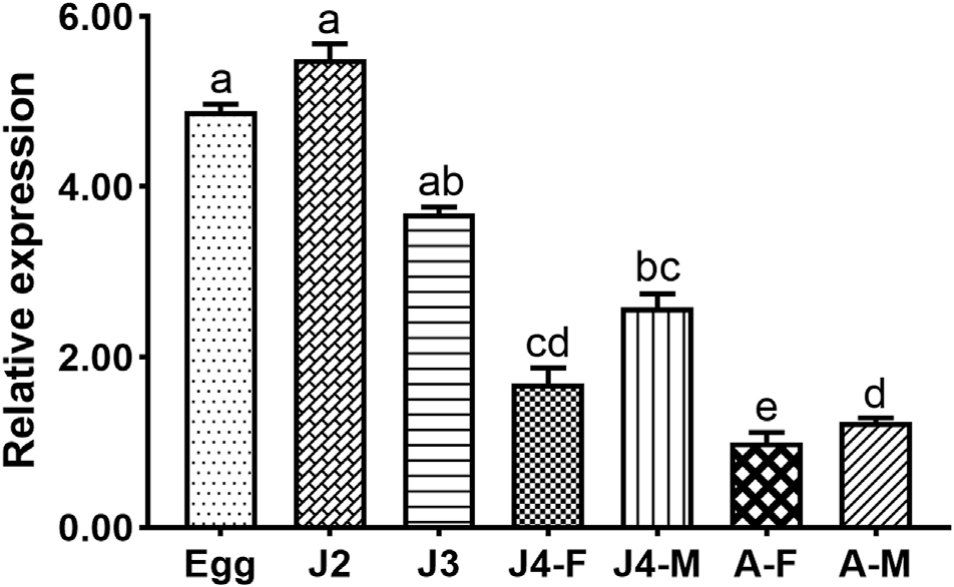
*Bxy-laf-1* expression levels in *B. xylophilus* at various developmental stages were investigated using real-time quantitative PCR. The *tbb-2* and *β-actin* endogenous reference genes were used to standardize the data. Adults were judged to have a 100% expression level. The same letter indicates that the difference between two groups is not significant, whereas distinct letters indicate that the difference between two groups has reached a significant level (*p* < 0.05). The standard error of the mean is shown by the error bars. Adult, sexually mature worms; Egg, the embryo-stage; J2, the second-stage juveniles; J3, the third-stage juveniles; J4-F, the fourth-stage of female juveniles; J4-M, the fourth-stage of male juveniles; A-F, the adult stage of female juveniles; A-M, the adult stage of male juveniles.

The expression profiles of *Bxy-laf-1* were investigated at different stages including embryonic stages, juveniles (J2, J3, J4), and adult stages in *B. xylophilus*. In the early ontogenesis of *B. xylophilus*, the expression level of *Bxy-laf-1* increased, and reached its highest level at the J2 stage (Figure 4). Subsequently, the expression level of *Bxy-laf-1* gradually decreased from the J3 stage and reached its lowest level at the adult stage, the gene expression level of males was significantly higher than that of females, and the gene expression pattern of females and males was different. These results suggest that *Bxy-laf-1* gene may play an important role in embryonic and J2 stages of *B. xylophilus*.

### 3.4 Spatial-temporal expression of *Bxy-laf-1*

**FIGURE 5:**
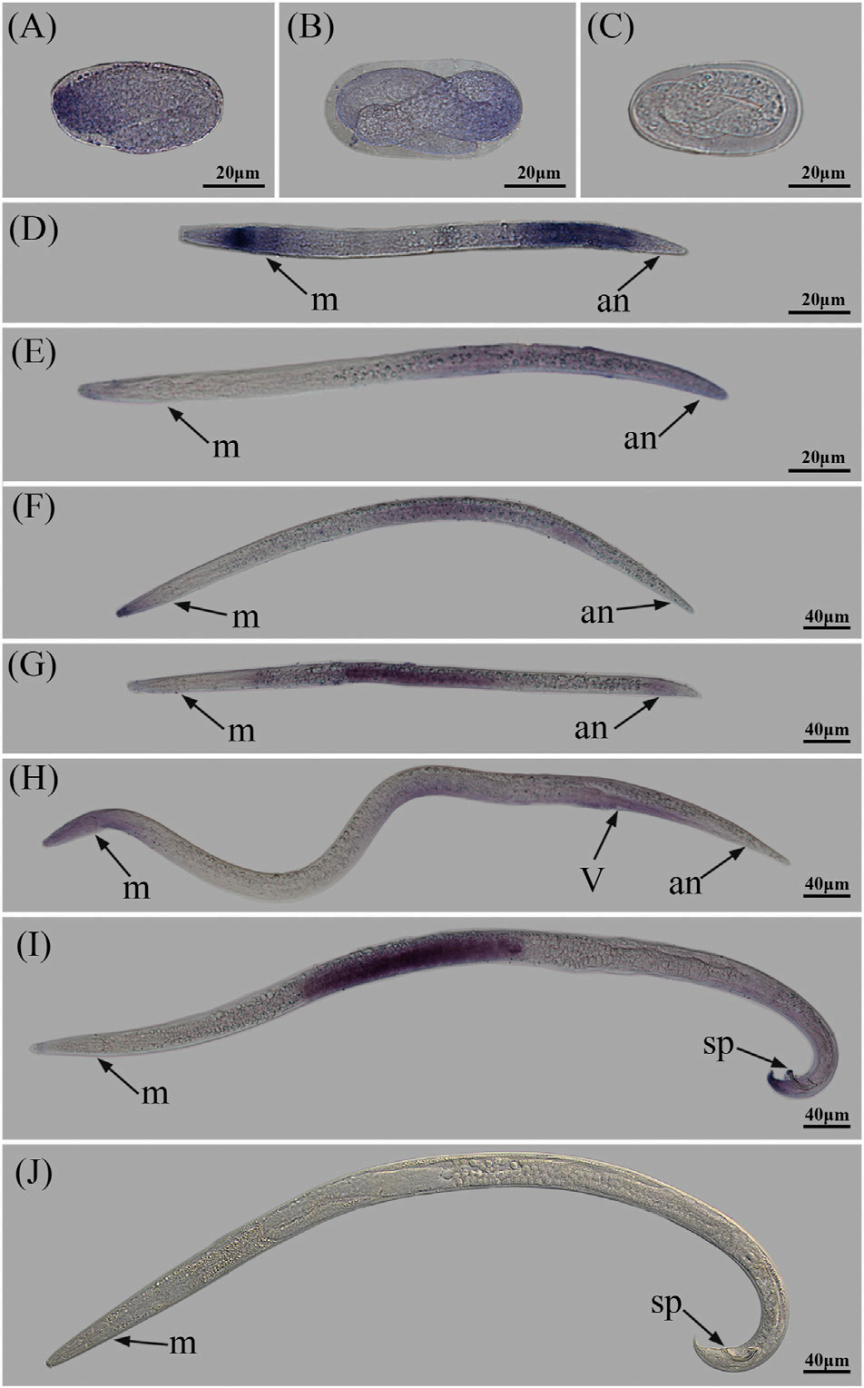
Localization of *Bxy-laf-1* mRNA in *B. xylophilus via in situ* hybridization. Hybridization with the digoxigenin-labelled specific probe in embryo **(A,B)**, no staining was observed in embryo **(C)**, second-stage juvenile **(D)**, third-stage juvenile **(E)**, female fourth-stage juvenile **(F)**, male fourth-stage juvenile **(G)**, female adult **(H)**, and male adult **(I)**. No staining was observed in male adult **(J)** with control sense probes. a, anterior; p, posterior; m, metacorpus; an, anus; v, vulva; sp, spicules.

Whole-mount *in situ* hybridization revealed that digoxigenin-labeled antisense probe staining was found from the early embryo to the adult, which was compatible with RT-qPCR findings. Single-ended staining is the expression pattern in the embryonic stage (Figures 5A,B). The stained region expanded as embryos developed. At the J2 and J3 stages, the staining began to centralize to the head region and tail region, which may be related to its motor activity (Figures 5D,E). The hybrid signal was observed in the gonads of male and female worms, as well as the portions that would develop as external sexual organs during the stage of sex differentiation (J4) (Ye and Feng, 1993) (Figures 5F,G). The hybridization signals were seen in the gonads and other reproductive organs of both male and female worms in the sexually mature adult stage. The staining in female adults was mostly located in the vulva, whereas the signal in male adults was mostly found around the tail spicules (Figures 5H,I). It is speculated that *Bxy-laf-1* may have an effect on the sex determination and mating behavior of *B. xylophilus*. In summary, it is speculated that *Bxy-laf-1* was predominantly expressed in the head, gonads, and sex organs of *B. xylophilus*.

### 3.5 Multiple roles of *Bxy-laf-1* in *B. xylophilus*

#### 3.5.1 Efficiency of RNA interference

**FIGURE 6:**
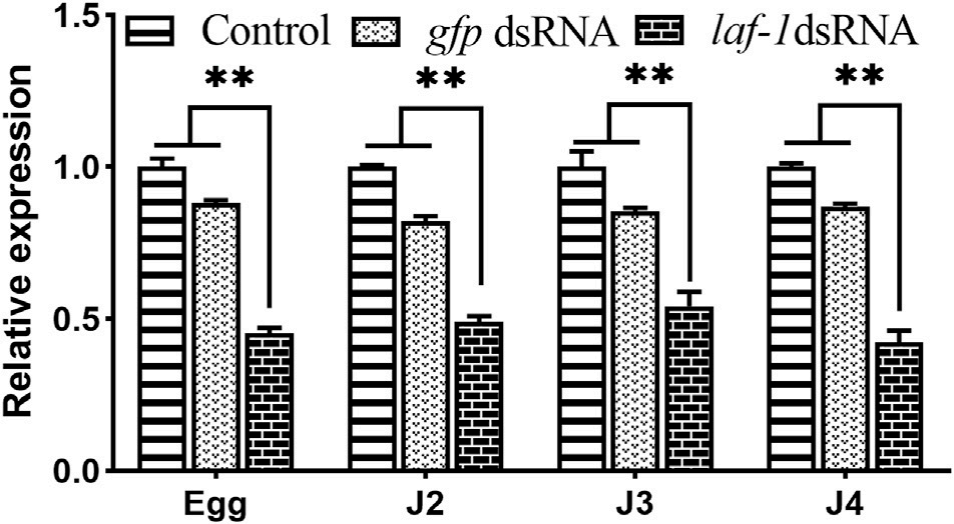
Real-time quantitative PCR analysis of *Bxy-laf-1* expression levels after RNAi soaking at different developmental stages in *B. xylophilus*. Egg, the embryo-stage; J2, the second-stage juveniles; J3, the third-stage juveniles; J4, the forth-stage juveniles. Control, the no probe control, means that nematodes were treated with soaking buffer alone; *gfp* dsRNA, the negative control means that nematodes were treated with *gfp* dsRNA; *Bxy-laf-1* dsRNA, means that nematodes were treated with *Bxy-laf-1* dsRNA; Data were normalized to those of endogenous reference genes *tbb-2* and *β-actin*. Expression level of the control groups was considered as 100%. Asterisks indicate a significant difference between two groups (*means *p* < 0.05, **means *p* < 0.01). Error bars represent standard error of mean.

Three groups including the no probe control, *Bxy-laf-1* dsRNA treated, and foreign *gfp* dsRNA treated were utilized in this experiment. There was no statistically significant difference between the no probe control and *gfp* dsRNA-treated group (Figure 6). When compared to the control group, the relative expression levels of embryonic, J2, J3 and J4 stage synchronous *B. xylophilus* were down-regulated by *Bxy-laf-1* dsRNA interference, attaining a significant level difference (*p* < 0.05).

#### 3.5.2 *Bxy-laf-1* is essential for larva locomotion

The abnormal in sinusoidal locomotion was observed in all treated *B. xylophilus* larvae. In terms of movement morphology, the body of the J2 hatched from dsRNA-treated embryos curled up. The head slightly swayed, and the nematode lost the ability to move. The same phenomenon appeared in J2 and J3 larvae when treated with *Bxy-laf-1* dsRNA. This suggests that *Bxy-laf-1* may regulate the motor nerves of larvae.

**FIGURE 7:**
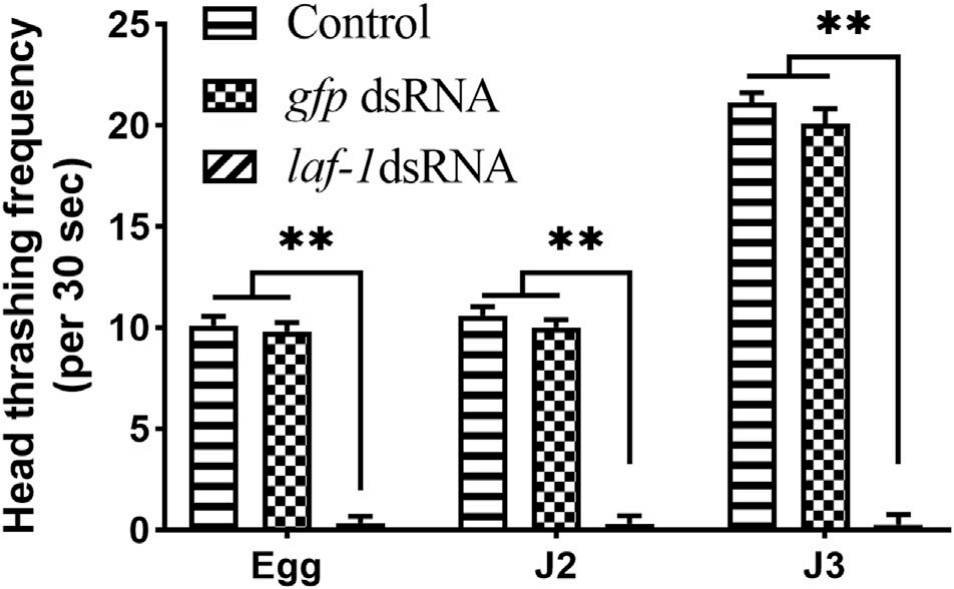
Head swing frequency of *B. xylophilus* after RNAi soaking at different developmental stages. Egg, larvae hatch from the treated embryo-stage; J2, the second-stage juveniles after 24 h treatment; J3, the third-stage juveniles after 24 h treatment. Control, the no probe control, means that nematodes were treated with soaking buffer alone; *gfp* dsRNA, the negative control means that nematodes were treated with *gfp* dsRNA; *Bxy-laf-1* dsRNA, means that nematodes were treated with *Bxy-laf-1* dsRNA. Asterisks indicate a significant difference between two groups (*p* < 0.05). Error bars represent standard error of mean.

In addition, a significant decrease in head swing frequency was observed in the larvae of all three stages mentioned above. In the *Bxy-laf-1* dsRNA treatment group, the head swing frequency of J2 hatched from the treated embryos was 0.33 ± 0.12 times 30 s^−1^ (*p* < 0.0005), and the treated J2 was 0.07 ± 0.06 times 30 s^−1^(*p* < 0.0005). J3 was 0.33 ± 0.12 times 30 s^−1^ (*p* < 0.0005). For the control groups, it was 10.10 ± 0.45 times 30 s^−1^, 10.60 ± 0.42 times 30 s^−1^, 21.13 ± 0.49 times 30 s^−1^, respectively (Figure 7). The frequency of head swing of *B. xylophilus* treated with *gfp* dsRNA was similar to that of the control treated with non-dsRNA. This demonstrates the importance of *Bxy-laf-1* in larval motility.

#### 3.5.3 *Bxy-laf-1* has effect on sex ratio of *B. xylophilus*

After treating embryos, J2, J3 and J4 with *Bxy-laf-1* dsRNA, the sex ratios of female to male were calculated. RNAi results showed that the F0 generation sex ratio was 1.63 ± 0.03 when treated during embryogenesis, and 2.07 ± 0.02 when treated at J2 compared to 2.7 around for the normal sex ratio. Statistical analysis also showed that both of them reached significant levels (*p* < 0.05). However, the sex ratio was 2.18 ± 0.03 when treated at J3 stage, or 2.47 ± 0.02 when treated at J4 stage.

**FIGURE 8:**
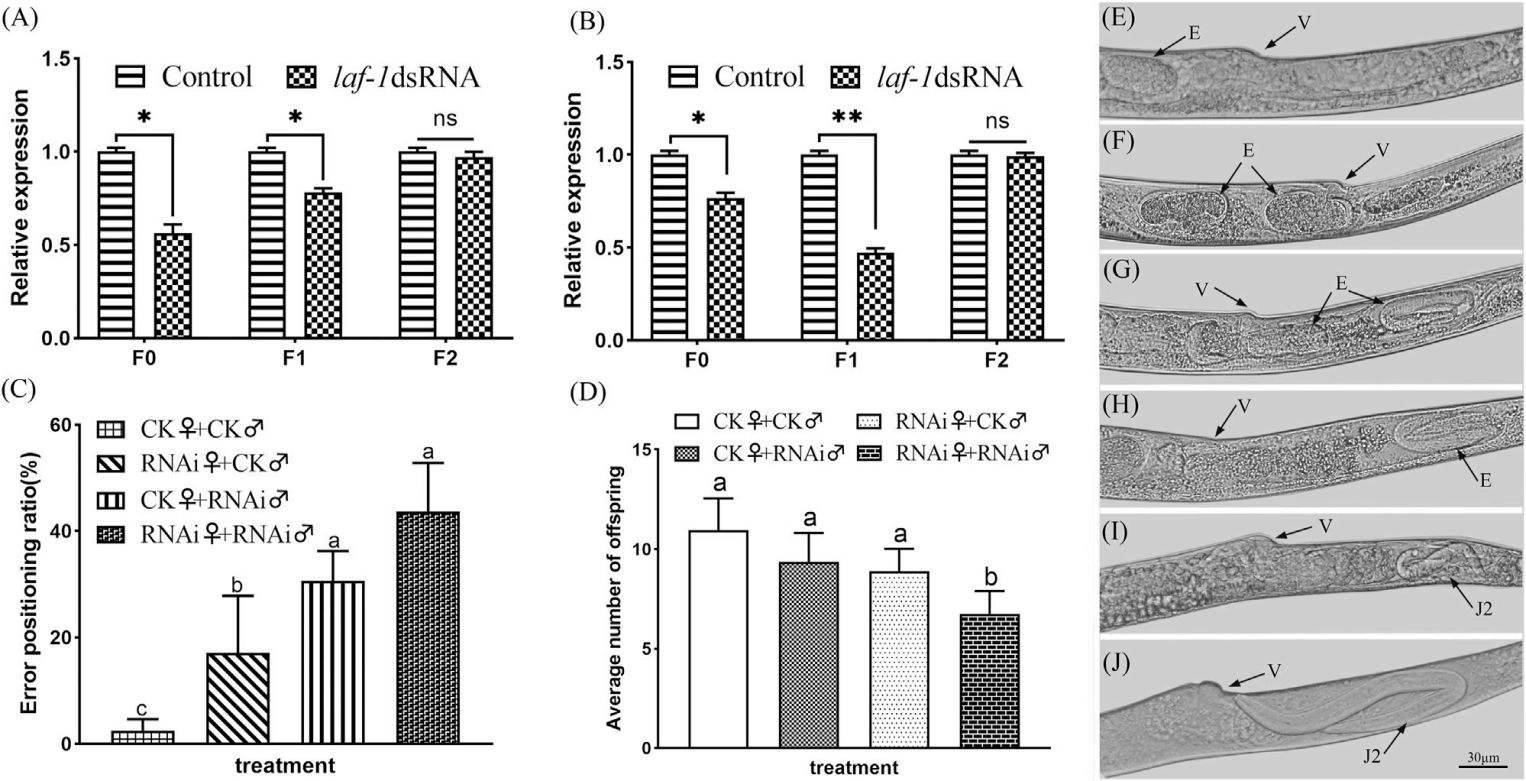
RNAi soaking by *Bxy-laf-1*. Real-time quantitative PCR analysis of *Bxy-laf-1* expression levels after RNAi soaking at embryos **(A)** and J2 **(B)**. Asterisks indicate a significant difference between two groups (*means *p* < 0.05, **means *p* < 0.01). **(C)** Error positioning ratio of *B. xylophilus* virgin adults. **(D)** Average number of offspring of *B. xylophilus*. The same letter indicates that the difference is not significant, and different letters indicate that the difference has reached a significant level between two groups (*p* < 0.05). **(E,F)** Position of embryos in the control group *in vivo*. **(G,H)** Embryos that have moved to the tail after *Bxy-laf-1* dsRNA treatment. **(I,J)** J2 larvae that have hatched in the tail. Error bars represent standard error of mean. v, vulva; E, embryo; J2, second-stage juveniles. Scale bar: 30 μm **(E–J)**.

The nematodes from the two stages in which the sex ratio decreased significantly were further cultivated to adults, and the sex ratio of the offspring was calculated. According to the RNAi results, the sex ratios of adult nematodes after embryonic and J2 treatments of *B. xylophilus* decreased to varying degrees in both the F0 and F1 generations, while the sex ratio of the F2 generation recovered to the normal level (Table 2). In order to verify the above results, the collected adult nematodes were tested for RNA interference efficiency. RT-qPCR results showed that the expression of *Bxy-laf-1* in F0 and F1 nematodes after RNAi was inhibited by *Bxy-laf-1* dsRNA to varying degrees (Figures 8A,B). These results indicated that knockdown of *Bxy-laf-1* has a certain degree of influence on the sex ratio of *B. xylophilus* and can last for one generation.

**TABLE 2:** The sex ratios in different groups of *B.xylophilus*.

Groups	Developmental stage	Sex ratio in adult nematodes (mean ± SE)		
		F0	F1	F2
RNAi Groups	Embryo	1.63 ± 0.03	1.85 ± 0.02	2.64 ± 0.03
	J2	2.07 ± 0.02	1.59 ± 0.03	2.65 ± 0.03
Control group		2.65 ± 0.04	2.63 ± 0.04	2.72 ± 0.04

#### 3.5.4 *Bxy-laf-1* participates in the reproduction and mating behavior of *B. xylophilus*

The error positioning ratio was significantly affected after being treated with *Bxy-laf-1* dsRNA. The error positioning ratio during the 8 h observation time was 3.33 ± 1.60% and 38.89 ± 3.96% for group of CK♀ + CK♂ and group of RNAi♀ + RNAi♂ respectively (*p* < 0.01) (Figure 8C). After the females or males were treated by *Bxy-laf-1* dsRNA, the males could not accurately locate the female vulva, resulting in an increase in the error positioning ratio. Subsequently, we placed nematodes in each group for 24 h after the end of observation, and counted the amount of eggs in each group. The average number of offspring after the 24 h was 10.94 ± 1.61 and 6.75 ± 1.14 for group of CK♀ + CK♂ and group of RNAi♀ + RNAi♂ respectively (*p* < 0.01) (Figure 8D). Based on the above phenomena, we observed and counted the characters of vulva parts of female worms after RNAi, and found that the embryos of adult female treated with *Bxy-laf-1* dsRNA did not emerge from the vulva but gradually moved through the vulva to the tail. The embryos located in the tail cannot be discharged from the body, and then hatched into J2, swimming in the female body, causing the female to die. This phenomenon occurred in both the F0 and F1 generations, with 14.3% of the F0 generation and 10.9% of the F1 generation affected.

## 4 Discussion

The laf-1 gene is a member of the DEAD box helicase family, that is highly consered from nematode to human. It plays an important role in sex determination and differentiation. This article introduces the functional and expression properties of the sequence.

### 4.1 *Bxy-laf-1* is evolutionarily conserved in *B. xylophilus*

*Bxy-laf-1* is evolutionarily conserved. Phylogenetic analysis showed that *Bxy-laf-1* and Bmu-LAF-1 clustered on the same branch and had similar homology with the model organism *C. elegans* LAF-1. Based on sequence alignment, three-dimensional structure and domains, it is further confirmed that *Bxy-laf-1* is evolutionarily conserved among species in different phyla.

### 4.2 *Bxy-laf-1* was mainly expressed in head, gonads and sexual organs of *B. xylophilus*

RT-PCR data showed that a basic level of *Bxy-laf-1* expression was needed throughout the life cycle of *B. xylophilus. Bxy-laf-1* was found in *B. xylophilus* embryos, tails of J2 and J3 stages (probably the portions that would develop into gonads), and gonads of J4 and adults, according to the results of mRNA *in situ* hybridization. In *C. elegans*, *laf-1* is an ortholog of *Bxy-laf-1*. It is expressed in the gonads of male *C. elegans* adults, which is quite similar to our findings in *B. xylophilus*. However, unlike *C. elegans*, *laf-1* is expressed at the highest level in the embryonic stage of *C. elegans* (Hubert and Anderson, 2009), while the highest level in *B. xylophilus* occurs in J2. Although the apparent structure of *C. elegans* and *B. xylophilus* is somewhat different, the expression pattern of *laf-1* in *C. elegans* is basically consistent with that of *Bxy-laf-1* in *B. xylophilus*.

### 4.3 Biological functions of *Bxy-laf-1* in *B. xylophilus*

Successful knockdown of *Bxy-laf-1* in *B. xylophilus* embryos and J2 resulted in obvious abnormal phenotypes. Impairment in locomotion indicates possible deficits in the muscle or neurons. *laf-1* gene belongs to DEAD-box protein family. Coincidentally, related genes of the DEAD-box protein family have been shown to regulate motor functions in insects and vertebrates. For instance, the loss of Gemin3 in the muscles of *Drosophila* larvae can cause the flight muscles to degenerate and lose the ability to fly (Cauchi et al., 2008; Shpargel et al., 2009). The mutation of *ddx39 ab* in zebrafish can cause trunk muscular dystrophy (Zhang et al., 2018). We therefore speculate that *Bxy-laf-1* is related to larval muscle development and affects the movement of *B. xylophilus* larvae.

The lack of *Bxy-laf-1* significantly reduced the female to male sex ratios of *B. xylophilus*. Related genes of the DEAD-box family are involved in sex differentiation in *C. elegans* (Hubert and Anderson, 2009), *zebrafish* (Sone et al., 2020) and *channel catfish* (Tian et al., 2017). Our results found that after *Bxy-laf-1* dsRNA interfered with *B. xylophilus*, the ratio of female to male in F0 and F1 generations was significantly reduced, indicating that *Bxy-laf-1* is involved in the development of sexual differentiation in *B. xylophilus*. In *C. elegans*, the sex determination pathway is regulated by multiple genes (Simone et al., 2012), the *laf-1* gene is located upstream of *tra-2* promoting female development (Doniach, 1986). It affects the export of *tra-2* mRNA from the nucleus by encoding DEAD-box RNA helicase, thereby promoting the ontogeny of *C. elegans* males. Our results indicate that the *laf-1* gene promotes the female ontogeny of *B. xylophilus*, which is contrary to the results of the model organism *C. elegans*. Further studies have shown that there is no homologous *tra-2* gene in *B xylophilus*. From this we speculate that *Bxy-laf-1* directly suppresses the expression of *fem* promoting male development (Doniach and Hodgkin, 1984) genes downstream of *tra-2*, thereby promoting female ontogeny. Here, we preliminarily speculated the function of *Bxy-laf-1* in the sex determination pathway of *B xylophilus*. The relationship between *Bxy-laf-1* and other sex determination genes requires further research.

After RNAi, the number of eggs laid by the females decreased significantly, about 14% and 11% of female’s embryos in F0 and F1 moved to the tail and could not be layed successfully. Studies have shown that mutations in genes in the DEAD-box protein family in *C. elegans* can cause the gonads to shrink (Ryuji et al., 2009). At the same time, it regulates the development of the gonads in both *Romanomermis wuchangensis* and *Ovomermis sinensis* (Duan et al., 2017; Tao et al., 2018). In addition, related genes in the DEAD-box family have similar regulatory effects on insects and mammals. For example, the knockdown of DDX3 and DDX6 in *L. migratoria* can impair ovarian development and oocyte maturation (Wang et al., 2021a; Wang et al., 2021b). In *Drosophila*, 12 genes are known to encode DEAD-box protein (Consortium, 2003), and the functions of *bel* and *vasa* are necessary for female fertility (Hay et al., 1988; Lasko and Ashburner, 1988; Styhler et al., 1998; Johnstone et al., 2005). We speculate that the knockdown of the *Bxy-laf-1* may cause abnormal ovarian development in *B. xylophilus* females, causing the embryos to move abnormally to the tail. In addition, in our search results, the positioning error rate of males in mating behavior increased sharply after interference. The lack of Dhh1 in *Saccharomyces cerevisiae* directly leads to serious mating defects (Ka et al., 2008). Therefore, it can be inferred that *Bxy-laf-1* also has an effect on mating behavior.

It has been suggested that sex determination of *Bursaphelenchus* nematodes may not be genome-base or environmental-base (Shinya et al., 2022). In recent years, the sex-determining genes of *B. xylophilus* have been extensively studied. For example, *mab-3* is essential for spermatogenesis in *B. xylophilus* (Zhou et al., 2020). In addition, more and more evidence indicates that the sex determination mechanism of *B. xylophilus* is also affected by environmental factors, such as nutrient conditions (Wei et al., 2021) and volatile substances concentration (Cui et al., 2018). Therefore, there may be multiple factors in the sex determination mechanism of *B. xylophilus*, which need to be further explored.

## 5 Conclusion

It is well known that *C. elegans* laf-1 is required for somatic sex determination. Our results show that *B. xylophilus* laf-1 was also required for sex differentiation. In two generations, knocking down *Bxy-laf-1* expression resulted in a drop in the sex ratio of female to male and impacted female ovarian development. In addition, *Bxy-laf-1* played an important role in mating behavior. The findings may contribute to a better understanding of the molecular mechanisms of sex determination and differentiation in this invasive worm, as well as give hopeful ideas for controlling pine wilt disease by preventing ontogenesis and limiting nematode reproduction.

## Data Availability

The datasets presented in this study can be found in online repositories. The names of the repository/repositories and accession number(s) can be found below: https://www.ncbi.nlm.nih.gov/genbank/, OM210018.
